# Reviewed article: Lima GCC, Passos CM, Pinheiro ALS, Ribeiro IJS, Maia EG. Temporal trend and epidemiological profile of notifications of violence against women in Brazil: 2014 to 2023. Epidemiol Serv Saude.2025:34;e20240475.

**DOI:** 10.1590/S2237-96222025v34e20240475.a

**Published:** 2025-08-08

**Authors:** Edgar Oshiro

**Affiliations:** Secretaria de Estado de Saúde de Mato Grosso do Sul, Escola de Saúde Pública

**Table:** 

Rating	Excellent	Good	Average	Below average	Poor
Interest	✓				
Quality		✓			
Originality			✓		
Overall		✓			

**Table:** 

Questionnaire	Yes	No	Not applicable
Does the manuscript contain new and significant information to justify publication?	✓		
Does the Abstract (Summary) clearly and accurately describe the content of the article?	✓		
Is the problem significant and concisely stated?	✓		
Are the methods described comprehensively?	✓		
Are the interpretations and conclusions justified by the results?	✓		
Is adequate reference made to other work in the field?	✓		

## Manuscript Structure

Length of article is: Adequate

Number of tables is: Adequate

Number of figures is: Adequate

Please state any conflict(s) of interest that you have in relation to the review of this paper (state “none” if this is not applicable): None

## Comments

O manuscrito apresenta uma estrutura clara e organizada, porém sugere-se uma revisão da redação no último parágrafo dos Resultados e da Discussão na questão do encaminhamento e da evolução do caso para uma melhor compreensão do assunto. 

